# Tunable quantum interferometer for correlated moiré electrons

**DOI:** 10.1038/s41467-023-44671-4

**Published:** 2024-01-09

**Authors:** Shuichi Iwakiri, Alexandra Mestre-Torà, Elías Portolés, Marieke Visscher, Marta Perego, Giulia Zheng, Takashi Taniguchi, Kenji Watanabe, Manfred Sigrist, Thomas Ihn, Klaus Ensslin

**Affiliations:** 1 Laboratory for Solid State Physics, ETH Zurich, CH-8093 Zurich, Switzerland; 2 Research Center for Materials Nanoarchitectonics, National Institute for Materials Science, 1-1 Namiki, Tsukuba, 305-0044 Japan; 3 Research Center for Electronic and Optical Materials, National Institute for Materials Science, 1-1 Namiki, Tsukuba, 305-0044 Japan; 4 Institute for Theoretical Physics, ETH Zurich, CH-8093 Zurich, Switzerland; 5 Quantum Center, ETH Zurich, CH-8093 Zurich, Switzerland

**Keywords:** Superconducting devices, Electronic properties and devices

## Abstract

Magic-angle twisted bilayer graphene can host a variety of gate-tunable correlated states – including superconducting and correlated insulator states. Recently, junction-based superconducting moiré devices have been introduced, enabling the study of the charge, spin and orbital nature of superconductivity, as well as the coherence of moiré electrons in magic-angle twisted bilayer graphene. Complementary fundamental coherence effects—in particular, the Little–Parks effect in a superconducting ring and the Aharonov–Bohm effect in a normally conducting ring – have not yet been reported in moiré devices. Here, we observe both phenomena in a single gate-defined ring device, where we can embed a superconducting or normally conducting ring in a correlated or band insulator. The Little–Parks effect is seen in the superconducting phase diagram as a function of density and magnetic field, confirming the effective charge of 2*e*. We also find that the coherence length of conducting moiré electrons exceeds several microns at 50 mK. In addition, we identify a regime characterized by *h*/*e*-periodic oscillations but with superconductor-like nonlinear transport.

## Abstract (web summary)

Gate-defined superconducting moiré devices offer high tunability for probing the nature of superconducting and correlated insulating states. Here, the authors report the Little–Parks and Aharonov–Bohm effects in a single gate-defined magic-angle twisted bilayer graphene device.

## Introduction

Magic-angle twisted bilayer graphene (MATBG) with its moiré flat band^1,2^ constitutes a condensed-matter system to realize a wide variety of correlated states, such as superconducting and correlated insulator states, that are tunable by gating^3–9^. A class of gate-defined nanodevices, including Josephson junctions^10,11^ and SQUIDs^12^, have been recently realized in MATBG. These structures have provided excellent platforms for controlling mesoscopic superconductivity and characterizing MATBG. Extending this approach to a doubly-connected geometry without any junction, namely a ring, promises unique microscopic information about the material and the device.

The physical properties of a ring threaded by a magnetic field are in general periodic in flux quanta^13,14^ ${\Phi }_{0}=\frac{h}{{e}^{*}}$, with e^*^ being the charge of the carrier. In a superconducting ring (e* = 2*e*), *h*/2*e*-periodic oscillations of critical temperature and critical current appear. These oscillations are known as the Little–Parks effect and were the first experimental evidence for the 2*e* charge pairing in conventional superconductors^15,16^. In fact, the Little–Parks effect can be used to determine the charge of the superconducting carriers^17,18^, complementing the Josephson junction and SQUID experiments^10,12^. Moreover, properties of unconventional superconductors can be revealed by anomalies of the Little–Parks effect, such as a phase shift^19–24^ or a change in periodicity^24–29^, and thereby help to understand the underlying superconducting symmetry.

By contrast, a normally conducting ring (e* = *e*) shows *h*/*e*-periodic oscillations of resistance, the Aharonov–Bohm effect, and works as a direct probe to quantify the phase coherence of electrons. Given the low Fermi velocity and the large effective mass in MATBG, a possible non-Fermi liquid nature of its flat band electrons^4,30^, and intrinsic disorders introduced by twist-angle inhomogeneity^31^, quantifying the phase coherence length is key to understanding the dynamics of moiré electrons. In addition, the phase coherence length enables the estimation of the penetration depth of a superconducting wave function into the normally conducting state (proximity effect)^32,33^, which plays an important role in gate-defined superconducting devices. However, the exploration of these fundamental quantum-interference effects has been hampered by the lack of a suitable device architecture and the sensitivity of the moiré superlattice to disorder^31^, which poses a challenge to the conventional approach of fabricating a ring by physical/chemical etching.

Here, we report the observation of Little–Parks and the Aharonov–Bohm effects in MATBG. The device architecture allows us to define a ring consisting of a loop that can be tuned to be superconducting or normally conducting, surrounded by a correlated or band insulator. We confirm 2*e* pairing via the Little–Parks effect, and show that the phase coherence length of moiré electrons surpasses several microns at 50 mK, evidenced by *h*/*e*-periodic Aharonov–Bohm oscillations. We also discover an intriguing regime in which *h*/*e*-periodic oscillations appear alongside superconductor-like transport. These results highlight the promise of the quantum interferometer in MATBG for studying interference phenomena of exotic quantum states of 2D materials.

## Results

### Highly tunable quantum interference

**Fig. 1:**
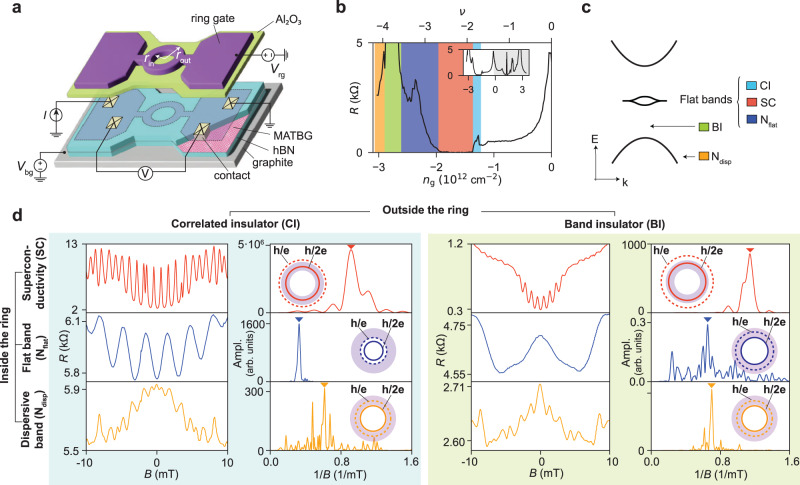
Highly tunable quantum interference. **a** Device and measurement schematics. The graphite–hBN–MATBG–hBN heterostructure is contacted by four electrodes (yellow). The ring gate (purple) is formed on top of an aluminium oxide layer (green). The lithographic inner (*r*_in_ = 600 nm) and outer (*r*_out_ = 1000 nm) radius of the ring gate are indicated. DC-voltage sources are connected to the ring gate and back gate. A four-terminal measurement is performed by applying a constant current and measuring the voltage drop across the ring. **b** Resistance of the MATBG as a function of carrier density *n*_g_ at ring (top) gate voltage *V*_rg_ = 0 V at 50 mK. The regions with colour background correspond to the density ranges of the states introduced in (**c**). The inset shows the resistance for both the electron (grey background) and hole side (white background). A background resistance of 130 Ω is subtracted from the resistance trace. **c** Band structure schematics of MATBG. The colour-coded labels indicate the correlated insulator (CI), superconducting (SC), normally conducting in the flat band (N_flat_), normally conducting in the dispersive band (N_disp_) and band insulator (BI) states, respectively. **d** Overview of quantum-interference effects. A ring-shaped conducting path is defined by surrounding the ring with a CI (left panel, blue-shaded) or BI (right panel, green-shaded) state. For both insulating states outside the ring, the magneto-resistance oscillations and their FFT spectrum are shown for the three conducting states: SC (top row), N_flat_ (middle row) and N_disp_ (bottom row). The triangle in the spectrum marks the peak frequency. The insets show the effective radius of the ring for both *h*/2*e* and *h*/*e* oscillations (solid and dashed circles, respectively), on top of the lithographic area of the ring gate (purple).

We develop the gate-defined ring architecture shown in Fig. 1a. We base the design on the proof-of-principle device reported in ref. ^34^ using Bernal bilayer graphene. The MATBG is encapsulated in hexagonal boron nitride (hBN) and is contacted by four electrodes. The sample is dual-gated with a graphite back gate and a metallic ring-shaped top gate (ring gate). We operate the ring by first tuning the back gate voltage *V*_bg_, which affects the entire MATBG area and induces a global density *n*_g_. Then we tune the density under the ring gate, *n*_r_, via the voltage *V*_rg_. The ring has a lithographic inner radius of *r*_in_ = 600 nm and outer radius of *r*_out_ = 1000 nm. Through the electrodes, the sample is biased with a current *I*, and the voltage drop *V* is measured in a four-terminal configuration. Unless stated otherwise, the measurements are performed in a ^3^He–^4^He dilution refrigerator at a temperature of 50 mK.

The resistance *R* of the MATBG (Fig. 1b) as a function of *n*_g_ with *V*_rg_ set to zero shows pronounced peaks at charge neutrality, and in the correlated insulator (CI) and band insulator (BI) regimes on the hole side (*n*_g_ < 0). From the density at the BI peak, we estimate an average twist angle of 1.1^∘^. When tuning the density beyond the correlated insulator, we observe superconductivity (SC) through a resistance drop. We also access two normally conducting regimes: one inside the flat band (*N*_flat_) and the other in the dispersive band (*N*_disp_). Figure 1c summarizes the relevant quantum states that form in the device. Further details of the experimental setup are given in the Methods section.

In order to define a conducting path, we tune the ring-shaped region into the SC, N_flat_, or N_disp_ regimes. This conducting path is then surrounded by either CI or BI states to confine electrons. As we will see below, the insulating state does not influence the observed interference pattern but has an effect on the quantum state distribution across the structure (see Supplementary Fig. 8). Figure 1d shows the resistance (*R*) oscillations in perpendicular magnetic field (*B*) for the six regimes at zero bias current and a temperature of 150 mK, together with their fast Fourier transform (FFT) spectra. When calculating the FFT spectrum, we subtract a smooth background extracted with the Savitzky–Golay filter (smoothing window of 10 mT and polynomial order 2). In this process, long-period signal in magneto-resistance, such as the universal conductance fluctuations, is filtered out. We convert the peak of the spectrum into an area assuming either *h*/*e* or *h*/2*e*-periodicity as the relevant flux quantum and then compare the result with the lithographic radius of the ring (see inset circles in Fig. 1d).

**Fig. 2:**
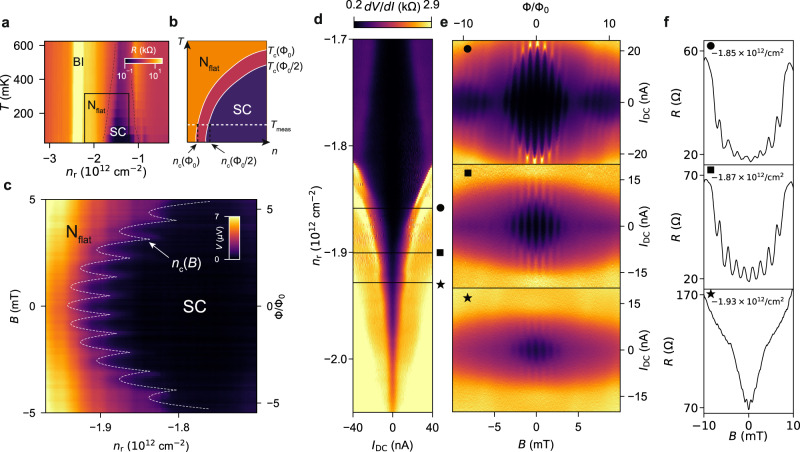
Tunable Little–Parks oscillations. **a** Phase diagram of the device in the (SC, BI) configuration. The SC, N_flat_ and BI states are represented. The square indicates the (*T*, *n*) domain for which we sketch the schematics in (**b**). **b** Schematics of the superconducting dome in the phase diagram of MATBG when the flux threading the ring is Φ = *n*Φ_0_ (denoted as Φ_0_) and $\Phi=\frac{n}{2}{\Phi }_{0}$ (denoted as Φ_0_/2), where Φ_0_ is the superconducting flux quantum. *T*_c_(Φ_0_) and *T*_c_(Φ_0_/2) mark the boundary between the N_flat_ (orange) and the SC dome (in pink for Φ_0_ and purple for Φ_0_/2). Upon applying a magnetic field, the phase diagram oscillates between *T*_c_(Φ_0_) and *T*_c_(Φ_0_/2). **c** Voltage drop *V* across the ring as a function of *n*_r_ and *B*, at *I* = 5.0 nA. The white dashed curve (a guide to the eye) marks the phase boundary *n*_c_(*B*) between the SC and N_flat_ states. **d** $\frac{dV}{dI}$ as function of ring density *n*_r_ and DC current *I*_DC_ when defining the ring in the BI state with *n*_g_ = − 2.94 × 10^12^ cm^−2^. **e** Magneto-resistance oscillation of the critical current taken at the densities indicated in **d** with a black circle, square, and star, respectively. **f** Magneto-resistance oscillations taken at *I*_DC_ = 0 nA and at the same densities as in (**e**).

In the case of a superconducting ring (top row in Fig. 1d, *n*_r_ = − 1.89 × 10^12^ cm^−2^), the frequency peak appears at 0.92/mT for (SC, CI) and at 1.20/mT for (SC, BI), respectively. Hereafter, we denote the state inside and outside the ring as (inside, outside). Assuming *h*/2*e*-periodicity, the observed frequency peaks correspond to an effective radius *r*_eff_ of 767 nm and 855 nm, respectively. These values are comparable to the center-line radius of the ring gate ${r}_{{{{{{{{\rm{mid}}}}}}}}}=\frac{{r}_{{{{{{{{\rm{in}}}}}}}}}+{r}_{{{{{{{{\rm{out}}}}}}}}}}{2}=800\,{{{{{{{\rm{nm}}}}}}}}$. In contrast, the *r*_eff_ when assuming *h*/*e*-periodicity does not match the lithographic dimension of the ring gate (*r*_eff_ > 1000 nm = *r*_out_). In these regimes, we further observe critical current and critical density oscillations, as we discuss in Fig. 2. Based on our findings, we attribute these oscillations to the *h*/2*e*-periodic Little–Parks effect, confirming that the charge of the superconducting carrier is 2*e*.

When the ring is tuned into the dispersive band (bottom row in Fig. 1d, *n*_r_ = − 3.58 × 10^12^ cm^−2^), *h*/*e*-periodic oscillations appear with a spectrum covering a significant range in 1/*B*. The peak frequency of the oscillations is 0.621/mT for both (N_disp_, CI) and (N_disp_, BI). This frequency is approximately half of those observed in the superconducting case, suggesting an *h*/*e*-periodicity. The effective radii both assuming *h*/*e*- (*r*_eff_ = 873 nm) and *h*/2*e*-periodicity (*r*_eff_ = 617 nm) fit within the ring dimensions. However, the oscillation amplitude decays exponentially in temperature and strives at higher magnetic fields than the Little–Parks oscillations (see Supplementary Figure 2). The amplitude decay in temperature is characteristic of the Aharonov–Bohm oscillations due to the smearing of the Fermi function and the reduction of the phase coherence length^35^. We therefore attribute the oscillations to the *h*/*e*-periodic Aharonov–Bohm effect. The measurement of the temperature dependence also allows us to estimate the phase coherence length *L*_*φ*_ of electrons in the dispersive band (see Supplementary Fig. 2). At 50 mK, the coherence length is ~ 12.3 ± 0.3 μm in (N_disp_, CI) and ~18.7 ± 1.0 μm in (N_disp_, BI). These values exceed the perimeter of the ring (2*πr*_eff_ ≃ 4.80 μm). Now, we compare the phase coherence length of bilayer graphene in the literature. The coherence length *L*_*ϕ*_ of the ring of exfoliated Bernal (non-twisted) bilayer graphene encapsulated with hBN is 1.5 μm at 36 mK for the etching-defined ring^36^ and 4.4 *μ*m at 300 mK for the gate-defined ring^34^ (maximum values reported in each paper). In our experiment, *L*_*ϕ*_ of moiré electrons in MATBG exceeds 10 μm at 50 mK. Considering that the amplitude of the Aharonov–Bohm oscillations of these three reports is all proportional to 1/*T*, one can estimate (or normalize) the coherence length to that of 50 mK. Then, we obtain *L*_*ϕ*_ = 2.1, 26.4, and 10 μm, for the etched (Bernal bilayer), gate-defined (Bernal bilayer), and gate-defined (MATBG) ring, respectively.

This result demonstrates that the phase coherence of moiré electrons in the dispersive band is well preserved despite several sources of disorder such as twist-angle inhomogeneity and strain distribution.

**Fig. 3:**
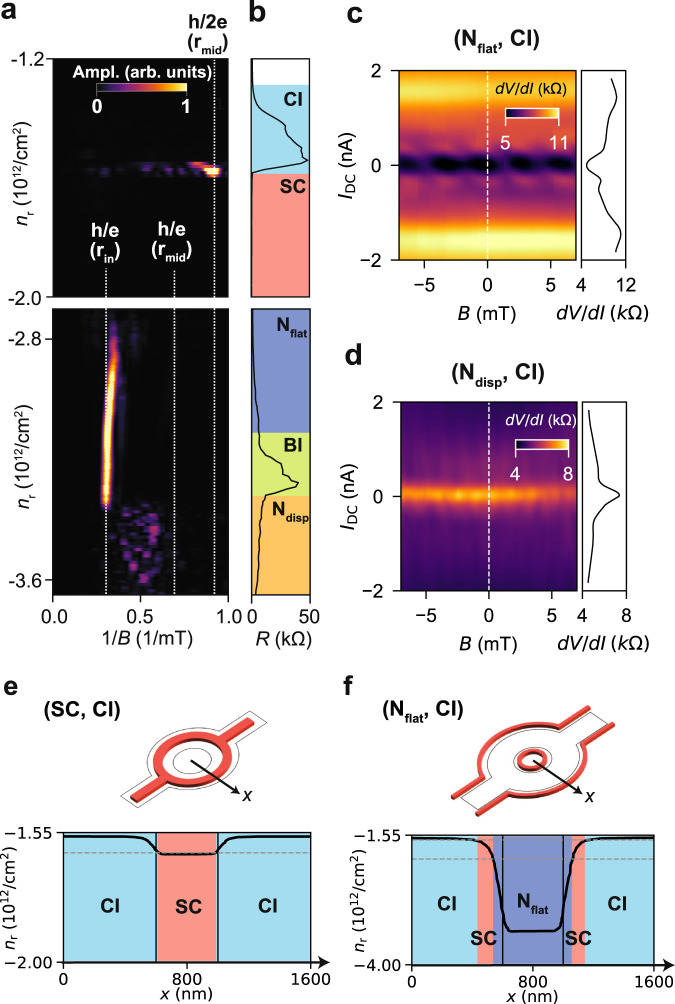
Spectroscopy of the quantum oscillations. **a** FFT spectrum of the magneto-resistance oscillations. The white lines in the spectrum indicate the range of frequencies expected for *h*/*e* and *h*/2*e* oscillations, taking the inner radius (*r*_in_) and mean radius (*r*_mid_) of the ring. **b** Resistance as a function of *n*_r_, the different quantum states (CI, SC, N_flat_, BI and N_disp_) are indicated. **c**, **d** $\frac{dV}{dI}$ as a function of *B* and *I*_DC_ **c**: in (N_flat_, CI) regime and **d**: in (N_disp_, CI) regime. In the side panel, linecut of $\frac{dV}{dI}$ at *B* = 0. **e**, **f** Electrostatic simulation of the carrier density and quantum state distribution **e**: in the (SC, CI) regime and **f**: in (N_flat_, CI) regime (**f**). The illustrations at the top show the superconducting regions (red) on the lithographic structure of the ring (black). At the bottom, plot of the spatial distribution of the carrier density along the radial axis of the ring (arrow in the illustration). The black vertical lines indicate the lithographic dimensions of the ring which is 400 nm. The horizontal grey dashed lines indicate the density range for superconductivity. Different quantum states (CI, SC, and N_flat_) are attributed depending on the density.

Furthermore, there is a striking contrast between the oscillation in the normally conducting flat band regimes depending on the surrounding insulators (middle row in Fig. 1d, *n*_r_ = − 3.10 × 10^12^ cm^−2^). In the (N_flat_, CI) regime, we observe an oscillation with a frequency of 0.630/mT, even lower than in the SC and N_disp_ cases. The effective radius assuming *h*/2*e* periodicity is *r*_eff_ = 432 nm, even smaller than *r*_in_, while *r*_eff_ = 605 nm for *h*/*e* periodicity matches *r*_in_. Though the geometric argument points towards *h*/*e* periodicity, this regime exhibits superconductor-like transport as well. We discuss this point in more detail later in Fig. 3. On the other hand, in the (N_flat_, BI) regime, we observe magneto-resistance oscillations with very small amplitude. In this regime, the frequency peak appears close to the one in the *N*_disp_ regime. The effective radius is *r*_eff_ = 813 nm assuming *h*/*e*-periodicity and *r*_eff_ = 585 nm assuming *h*/2*e*-periodicity. This regime shows neither nonlinear transport nor a drop in resistance with temperature. Therefore, we attribute the oscillations in the (N_flat_, BI) regime to *h*/*e*-periodic Aharonov–Bohm oscillations. The phase coherence length, estimated from the temperature dependence, is *L*_*φ*_ ~ 6.51 ± 1.32 μm, which is by a factor of 2–3 smaller than that for N_disp_. This relatively short *L*_*φ*_ in the flat band can be attributed to a large electron effective mass. In fact, the phase coherence length is proportional to the Fermi velocity *v*_Fermi_, which depends inversely on the effective mass m* (*L*_*φ*_ ∝ *v*_Fermi_ ∝ 1/m*), and the measured m^*^ in the flat and dispersive bands are different by a factor of 1–10^4^.

These results demonstrate that one can switch between the Little–Parks and the Aharonov–Bohm effects of MATBG by gate tuning. They also reveal the 2*e* charge pairing and long coherence lengths of the moiré electrons. In the following sections, we discuss each regime in more detail.

### Tunable Little–Parks oscillations

The Little–Parks effect is essentially the magneto-oscillation of the free energy of the superconducting state^37^, which results in the oscillation of the critical temperature *T*_c_ and the critical current *I*_c_. While the critical current is readily measurable, the current injection inevitably drives the system out of equilibrium, possibly giving rise to unwanted effects such as local breakdown of superconductivity^38–40^. Therefore, a measurement at equilibrium is preferable. However, measurement of the *T*_c_ oscillation is experimentally challenging as the expected amplitude is in the sub-mK range^37^. Here, taking advantage of the in-situ tunability of the carrier density in MATBG, we demonstrate an alternative route to detect Little–Parks oscillations. We probe the oscillation of *T*_c_ by translating it into the oscillation of the critical density *n*_c_ at which the superconducting transition occurs, enabling the detection of the Little–Parks effect near equilibrium. Figure 2a shows the phase diagram of the device in the (SC, BI) regime, measuring the resistance as a function of temperature *T* and *n*_r_ at zero magnetic field. Increasing the temperature from 50 mK to 600 mK, the density range of the SC state shrinks, forming a superconducting dome. Due to the Little–Parks effect, the *T*_c_ of the superconducting ring oscillates with the magnetic flux. As depicted in Fig. 2b, this results in a compression and expansion of the superconducting phase boundary. For a fixed temperature *T*_meas_, such breathing can be translated into an oscillation of the critical density *n*_c_, between *n*_c_(Φ = Φ_0_) and ${n}_{{{{{{{{\rm{c}}}}}}}}}(\Phi=\frac{1}{2}{\Phi }_{0})$. It is therefore possible to probe the magneto-oscillation of the phase boundary by fixing the temperature and sweeping the carrier density.

Figure 2c shows the measured voltage drop *V* across the ring as a function of *n*_r_ and *B*, at a DC bias current of 5 nA and in a density range close to the high-density edge of the superconducting dome (see Supplementary Figure 4 for the temperature and current dependence of the map). At a fixed *B*, a jump from zero to a finite voltage *V* marks the transition from the superconducting to the normally conducting regime. The magnetic field dependence of the density *n*_c_(*B*) represents the phase boundary. The shape of this boundary oscillates with a period of 0.87 mT, agreeing with an *h*/2*e*-periodicity (*r*_eff_ ≈ *r*_mid_). The smooth shift of the phase boundary with increasing *B* to higher electron densities reflects the shrinking of the superconducting dome in *B*. We observe the same result with a reversed current (−5 nA). The demonstration of the Little–Parks effect near equilibrium by constructing the phase diagram is one of the distinct advantages of the gate-defined architecture.

We can also track the development of the oscillations as the state departs from the vicinity of the SC-to-N_flat_ transition into deep inside the superconducting dome by tuning the density *n*_r_. Figure 2d shows *dV*/*dI* as a function of DC current *I*_DC_ and *n*_r_ at zero magnetic field, and Fig. 2e the *B*- and *I*_DC_-dependence of *dV*/*dI* for *n*_r_ = − 1.85, − 1.87 and − 1.93 × 10^12^ cm^−2^ (see solid horizontal lines in Fig. 2d). The periodic oscillations of the critical current *I*_c_ emerge on top of a decreasing background in magnetic field. The periodicity of the *I*_c_ oscillations is *h*/2*e* (0.87 mT), in agreement with the (SC, BI) data in Fig. 1d. As *n*_r_ is tuned to a value that shows larger *I*_c_ (deep inside the superconducting dome), the amplitude of the *I*_c_ oscillations increases, and they are also observed up to higher magnetic fields. Moreover, the magnetic field-origin of the oscillations shifts from zero and also depends on the direction of the applied current (see Supplementary Fig. 9), which can be due to the inductance of the ring. At the lowest density (Fig. 2e, top panel), we observe that the critical current vanishes at ~5 mT and re-emerges at a higher magnetic field. This pattern resembles the Fraunhofer pattern of Josephson junctions. However, as we discuss in the Supplementary Information section II.C, the width of this hypothetical Josephson junction does not fit any of our ring geometries. We therefore attribute the observed pattern rather to the existence of multiple interference paths within the ring, generating a beating pattern. Similar critical current oscillations but without a beating pattern appear in the (SC,CI) regime and are shown in Supplementary Fig. 5.

The differential resistance traces at zero bias current (*I*_DC_ = 0 nA) for the three densities are shown in Fig. 2f. Close to the SC-to-N_flat_ transition (bottom panel, star symbol), the magneto-resistance oscillations are barely visible as the difference of resistance between SC and the normal states is vanishing. When the density is increased (middle panel, square symbol), we observe pronounced oscillations where a chain of parabolas appears on top of a smooth parabolic background. When the density is further reduced (top panel, circle symbol), the oscillation amplitude drops again. This is because the state is deep in the superconducting dome and zero current is too remote from the transition to fully capture the Little–Parks effect. Deeper in the superconducting dome, one can only observe the critical current oscillations.

### Spectroscopy of the quantum oscillations

Figure 3a shows the magneto-oscillation spectrogram as a function of the density *n*_r_ when the state outside the ring is CI (see Fig. 1d, left column). The spectrogram is constructed by first measuring the differential resistance at zero bias current sweeping the magnetic field between ± 20 mT. Then the FFT spectrum is calculated after subtracting the smooth background from the raw data. In the spectrogram, we observe several regions with prominent peaks in the FFT. The resistance *R* at zero magnetic field and corresponding quantum states are shown in Fig. 3b.

An *h*/2*e* peak is observed (*r*_eff_ ≈ *r*_mid_) at the edge of the SC region in the vicinity of the CI regime. This can be understood by the fact that the Little–Parks oscillation only appears at the onset of the superconducting transition. When the density is tuned further down to the (*N*_flat_, CI) regime, a prominent peak at 0.313 /mT appears between −3.40 × 10^12^ cm^−2^ < *n*_r_ < −2.80 × 10^12^ cm^−2^. This corresponds to an effective radius of *r*_eff_ = 605 nm assuming *h*/*e*-periodicity, and *r*_eff_ = 432 nm assuming *h*/2*e*. As the latter *r*_eff_ is considerably smaller than *r*_in_, we attribute an *h*/*e*-periodicity to the oscillations. The density window across which this peak extends is unexpectedly wide and includes the BI regime, meaning that the oscillations persist even when the ring is mostly insulating. When entering the (N_disp_, CI) regime, the spectrum becomes broader and features a peak within the *h*/*e*-periodic range (*r*_in_ < *r*_eff_ < *r*_mid_). This broad spectrum is not unexpected for conventional Aharonov–Bohm oscillations as the aspect ratio of the gate-defined ring is small (*r*_in_/*r*_out_ ≃ 0.6), making possible many interference paths with different effective enclosed areas. The oscillations in (N_flat_, CI) and in (N_disp_, CI) not only differ in peak frequency and frequency extent but also show different responses to *I*_DC_. Figure 3c, d show the *I*_DC_ and *B* mapping in (N_flat_, CI), with *n*_r_ = 3.07 × 10^12^ cm^−2^, and (N_disp_, CI), with *n*_r_ = 3.51 × 10^12^ cm^−2^ respectively. Interestingly, a superconductor-like behaviour of the differential resistance (dip at *I*_DC_ = 0 nA) is observed in the (N_flat_, CI) regime, despite the high resistance reaching up to a few kΩ. In addition, a periodic chain of low-resistance states appears by sweeping *I*_DC_ and *B*. By contrast, the (N_disp_, CI) regime exhibits a cusp in differential resistance at *I*_DC_ = 0 nA, and no characteristic pattern is observed in the *I*_DC_ and *B* mapping. This again supports the interpretation that Aharonov–Bohm oscillations are observed for (N_disp_, CI).

We attribute the superconducting behaviour of the (N_flat_, CI) regime to the presence of a small residual superconducting region, emerging from the smooth evolution of the carrier density from inside to outside of the ring. To support this idea, we perform an electrostatic simulation of the carrier density distribution (see Supplementary Figure 8). As shown in Fig. 3e, in the (SC, CI) regime, SC is the only state inside the ring. However, when the ring is detuned from the (SC, CI) to the (N_disp_, CI) regime (Fig. 3f), the spatial distribution of the quantum states becomes complex, and a superconducting region can be embedded around the ring. This spurious superconducting region consists of a small ring with radius ~400 nm and a path surrounding the gated region, as Fig. 3f depicts.

The origin of the high visibility of the oscillations and its periodicity in the (N_flat_, CI) regime remains elusive, while careful inspection of the electrostatic environment might give an insight into it. For example, the spurious superconducting ring shown in Fig. 3f has a radius of ~400 nm, which is much smaller than the lithographic radius. However, it matches the effective radius of the oscillations in this regime, assuming *h*/2*e* periodicity (see Supplementary Fig. 8). This means that the spurious superconducting ring that is formed due to the electrostatic requirement might be giving the Little–Parks oscillations in the (N_flat_, CI) regime. Further improvement of the simulation, taking the proximity effect at the interface of different quantum states into account, will help the understanding of this regime.

## Discussion

In conclusion, we presented a gate-defined quantum interferometer in MATBG that provides a versatile platform for investigating the quantum coherence and the charge of correlated electrons from superconducting to normally conducting regimes. We observe the Little–Parks effect by constructing the superconducting phase diagram as well as by measuring the oscillation of the magneto-resistance and the critical current, confirming the charge-2*e* pairing. We also observe the Aharonov–Bohm effect for the dispersive and flat band electrons in the same device. From it, we find that the phase coherence length exceeds a few microns, highlighting its robustness. We find a regime that exhibits magneto-resistance and critical current oscillations even detuned from the superconducting regime, which might be due to the electrostatic constriction of the device.

These experiments demonstrate that exploring the Little–Parks effect has the potential to provide insight into both the charge and spin nature of the Cooper pair in MATBG. Notably, the measurement under an in-plane magnetic field along with the perpendicular field could enable the observation of phase shifts in Little–Parks oscillations. Such shifts could serve as indicators of unconventional pairings such as spin-triplet superconductivity^41^. Our observation of the Aharonov–Bohm oscillations and the estimation of the phase coherence length of the moiré electrons provide the experimental foundation for the understanding of Andreev reflection physics in exotic quantum interfaces such as superconducting v.s. Chern or Mott insulating interface. Moreover, the gate-defined architecture and the measurement scheme presented here can generally be implemented in other 2D superconductors (e.g., twisted multilayer graphene^42^, Bernal bilayer graphene^43^, and bilayer graphene/transition metal dichalcogenide^44^), opening up the path towards the direct quantification of charge, spin, and coherence of correlated electrons in a plethora of exotic quantum states.

## Methods

### Twist angle estimation

We extract the twist angle of the sample using the relation $\theta=2\arcsin \left(\frac{a}{2L}\right)$^3^. In this expression, *a* is the lattice constant of graphene and *L* is the moiré periodicity, which represents the distance between two adjacent AA-stacked regions. In turn, *L* is related to the area ${{{{{{{\mathcal{A}}}}}}}}$ of the moiré unit cell via $L=2\sqrt{2{{{{{{{\mathcal{A}}}}}}}}/\sqrt{3}}$. Within a moiré unit cell, four electrons can be accommodated due to spin and valley degeneracy. Then, the band insulator peak due to the twist appears at the electron density *n*_BI_ which corresponds to the occupation of 4 electrons per moiré unit cell ${{{{{{{\mathcal{A}}}}}}}}=\frac{4}{{n}_{{{{{{{{\rm{BI}}}}}}}}}}$. We obtain *n*_BI_ from the Landau fan and density mapping (see Supplementary Fig. 3). Our analysis yields an approximate twist angle of 1.11°.

### Device fabrication

The device stack is assembled using the dry pick-up method^45^. We exfoliate graphene and hexagonal boron nitride (hBN) flakes on a 285 nm p:Si/SiO_2_ wafer. We start by scratching a graphene flake in two using a tungsten needle with a tip diameter of 2 μm controlled by a micromanipulator. We pick up all the flakes using a polydimethylsiloxane/polycarbonate stamp. We first pick up the top hBN flake, with a thickness of 18 nm, at 90 °C. Then we proceed to pick up the graphene and assemble the twisted structure. For this, we first pick up half of the pre-cut graphene, rotate the microscope stage by 1.1° and then pick up the other half of the graphene, all at 40 °C. We encapsulate the graphene by picking up a bottom hBN flake, with a thickness of 55 nm. For the encapsulation, the stack is first contacted to the bottom hBN at 40 °C and the temperature of the stage is raised to 80 °C. Finally, we pick up a graphite flake of 29 nm at 100 °C that serves as a back gate. The stack is then deposited at 160 °C on a p:Si/SiO_2_ chip. After deposition, we clean the polycarbonate stamp using dichloromethane.

We contact the MATBG with edge contacts made by electron beam lithography followed by reactive ion etching, using CHF_3_/O_2_ (40/4 sccm, 60W). The contacts are then evaporated using Cr/Au (10/80 nm). We define the electrode lines in two steps using electron beam lithography and depositing Cr/Au (10/60 nm for the first step and 5/50 nm for the second). Then we etch the stack to define the mesa and deposit a 20 nm thick layer of aluminium oxide by atomic layer deposition. For the ring-shaped top gate, we again use electron beam lithography and evaporate Cr/Au (5/35 nm). The electrode line for the top gate is also done by electron beam lithography and evaporation using Cr/Au (10/70 nm). In Supplementary Figure 1 we show optical pictures of the different fabrication steps and an SEM image of a similar device.

### Measurement setup

We carry out all the measurements in a dilution refrigerator that uses a mixture of ^3^He and ^4^He with a base temperature of 55 mK. We apply a constant current bias between a pair of contacts across the ring and measure the voltage drop between another pair, also across the ring. To generate the bias current, we use an in-house-built d.c. source in series with a 100 MΩ resistor. We use a d.c. amplifier, also built in-house, and measure its output with a Hewlett Packard 3441A digital multimeter. Each gate is connected to a different voltage source of the same type as the one used for generating a d.c. current. We convert the voltages we apply to the gates to electron densities by a parallel plate capacitor model. We estimate the capacitance per unit area of the back and ring (top) gate to be *C*_bg_ = *ε*_0_*ε*_hBN_/*d*_bot_ and *C*_rg_ = *ε*_0_*ε*_hBN_*ε*_AlOx_/(*ε*_hBN_*d*_top_ + *ε*_AlOx_*d*_AlOx_), where *ε*_0_ is the vacuum permittivity, *ε*_hBN_ = 3.3 and *ε*_AlOx_ = 9.5 are the relative permittivities of the hBN and the aluminium oxide, *d*_top_ and *d*_bot_ are the thicknesses of the top and bottom hBN and *d*_AlOx_ is the thickness of the aluminium oxide layer. We calculate the electron density of the bulk as *n*_g_ = *C*_bg_*V*_bg_/*e* and of the region below the top gate as ${n}_{{{{{{{{\rm{r}}}}}}}}}=({C}_{{{{{{{{\rm{bg}}}}}}}}}{V}_{{{{{{{{\rm{bg}}}}}}}}}+{C}_{{{{{{{{\rm{rg}}}}}}}}}{V}_{{{{{{{{\rm{rg}}}}}}}}})/e$, where *e* is the elementary charge.

### Error/uncertainty analysis

Electronic noise that comes from the noise in the measurement equipment (e.g. amplifiers) was minimized by carefully removing the ground loops. The measured voltage was typically integrated by 200 ms to reduce the uncertainty of the data points. The error in the estimation of the coherence length was calculated from the standard deviation of the least-square fitting to the data and the error-propagation rule.

### Supplementary information

Supplementary Information

Peer Review File

## Data Availability

The data that support the findings of this study will be made available online through the ETH Research Collection at hdl.handle.net/20.500.11850/644891.

## Code availability

The code used for plotting the figures will be made available online through the ETH Research Collection at hdl.handle.net/20.500.11850/644891.
