# Supplementary material for: Tunable quantum interferometer for correlated moiré electrons
Source: Nat Commun. 2024 Jan 9;15:390. doi: 10.1038/s41467-023-44671-4 (PMC10776667; doi:10.1038/s41467-023-44671-4)
Supplement: Supplementary file 1 — Supplementary Information [file 41467_2023_44671_MOESM1_ESM.pdf]

Supplementary Information for: Tunable quantum interferometer for correlated moiré electrons

I. SUPPLEMENTARY FIGURES

A. Optical images and design of the sample

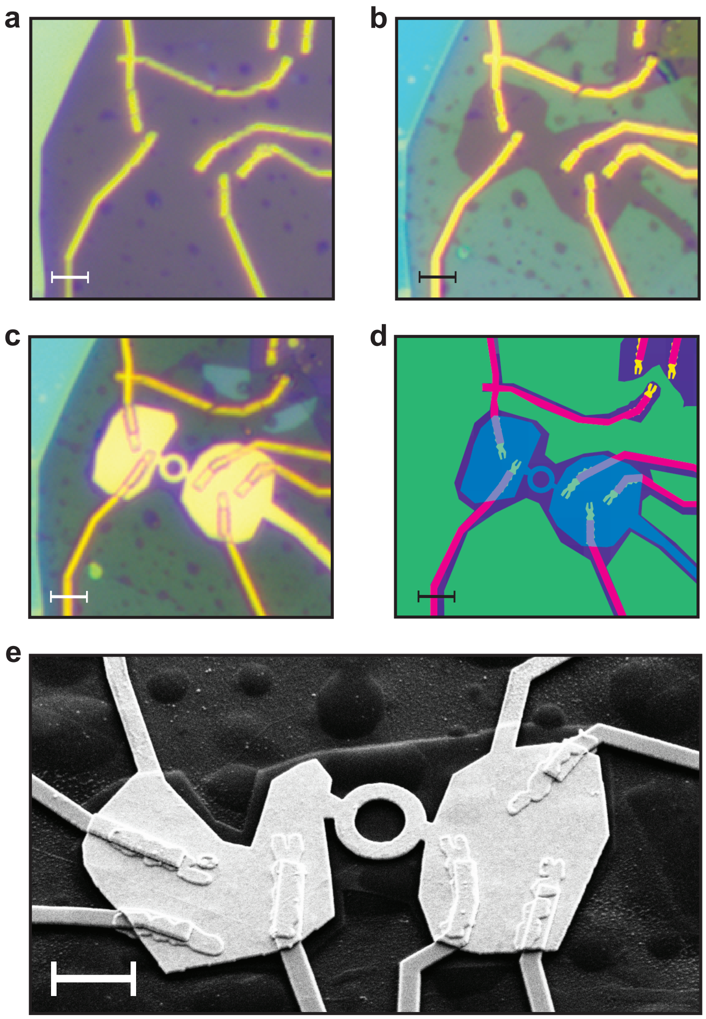

Supplementary Figure 1. **Optical images and design of the sample**. The device used in this work is designed and fabricated through a series of steps summarized in the Methods section. **a** Microscope picture of the device after forming contacts, **b** after etching the mesa and **c** after depositing a layer of aluminum oxide and the ring gate. **d** Design of the device. Contacts are represented in yellow, etched areas in green, and the ring gate in blue (half transparent). The scale bar in **a**-**d** is 5 µm. **e** SEM picture of the dummy device (not the one used in the measurement). The scale bar is 2 µm.

B. Magneto-resistance and its temperature dependence

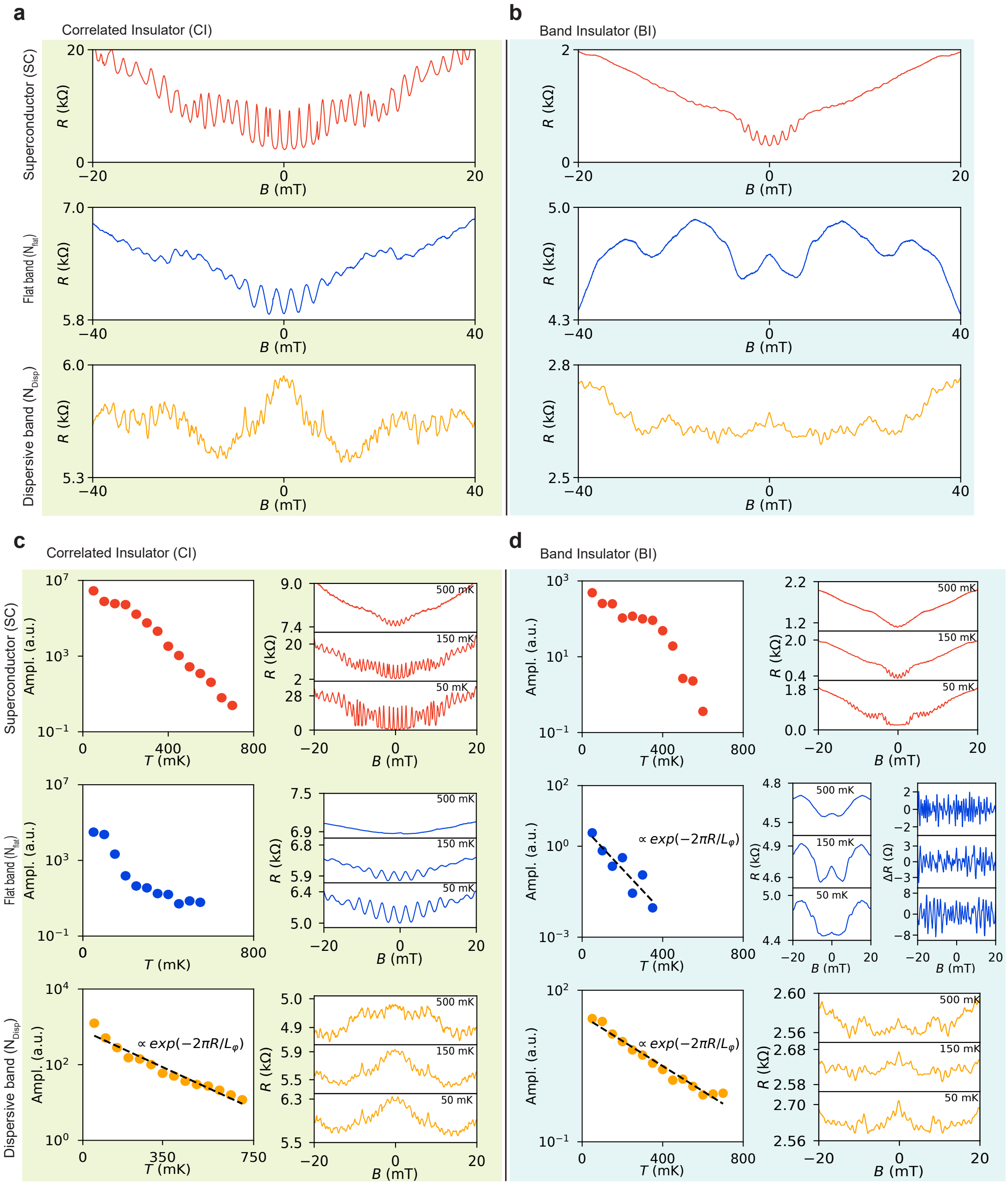

Supplementary Figure 2. Magneto-resistance and its temperature dependence. **a,b** Magneto-resistance traces discussed in Fig. 1d without background subtraction (raw data). **c,d** Temperature dependence of the oscillations (50, 100, 150, 200, 250, 300, 350, 400, 450, 500, 550, 600, 650, and 700 mK). The gate voltages for each configuration are the same as the ones in Fig. 2d. For the Aharonov–Bohm oscillations in (N_{disp}, CI) and (N_{disp}, BI) regimes, the temperature dependence is fitted with an exponential function $e^{-2\pi r_{eff}\alpha T}$, with r_{eff} being the effective radius of the ring estimated by the FFT peak frequency (temperature independent) and α is the coefficient that defines the temperature dependence of the phase coherence length $L_\phi = 1/\alpha T$. From this fitting, we obtain $L_\phi \sim 12.3 \pm 0.8$ µm for (N_{disp}, CI) regime, $L_\phi \sim 6.5 \pm 1.3$ µm for (N_{flat}, BI), and $L_\phi \sim 18.7 \pm 1.0$ µm for (N_{disp}, BI) regime.

C. Density maps and angle extraction of the sample

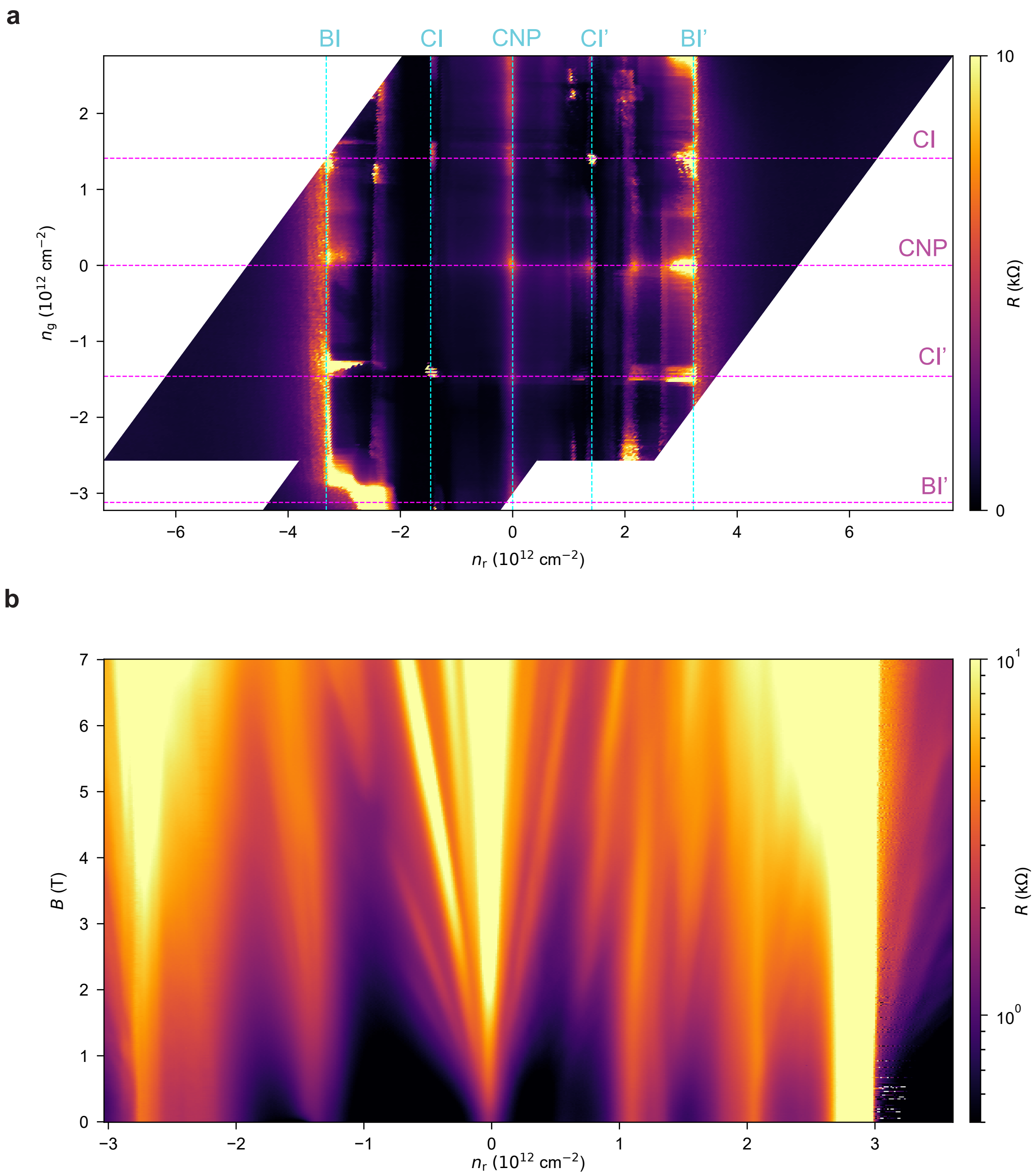

Supplementary Figure 3. **a** Resistance (R) across the device as a function of the carrier density controlled by the back gate (n_{g}) and the top gate (n_{r}). Densities are calculated from the capacitance model described in the Methods section. The dashed lines indicate the density values at which the charge neutrality point (CNP), CI and BI states arise in the region solely controlled by the back gate (pink) and in the top gated region (blue), both in the electron (with apostrophe) and hole (without) side. We use the BI densities to extract the twist angle of the device. **b** Magnetic field and n_{r} dependence of the resistance across the device (Landau fan) at $V_{\mathrm{rg}} = 0\,\mathrm{V}$. The measurement is taken at 2 K. Both measurements are taken using an AC current of 1 nA.

D. Critical density oscillations in the CI regime for different DC-bias currents and temperatures

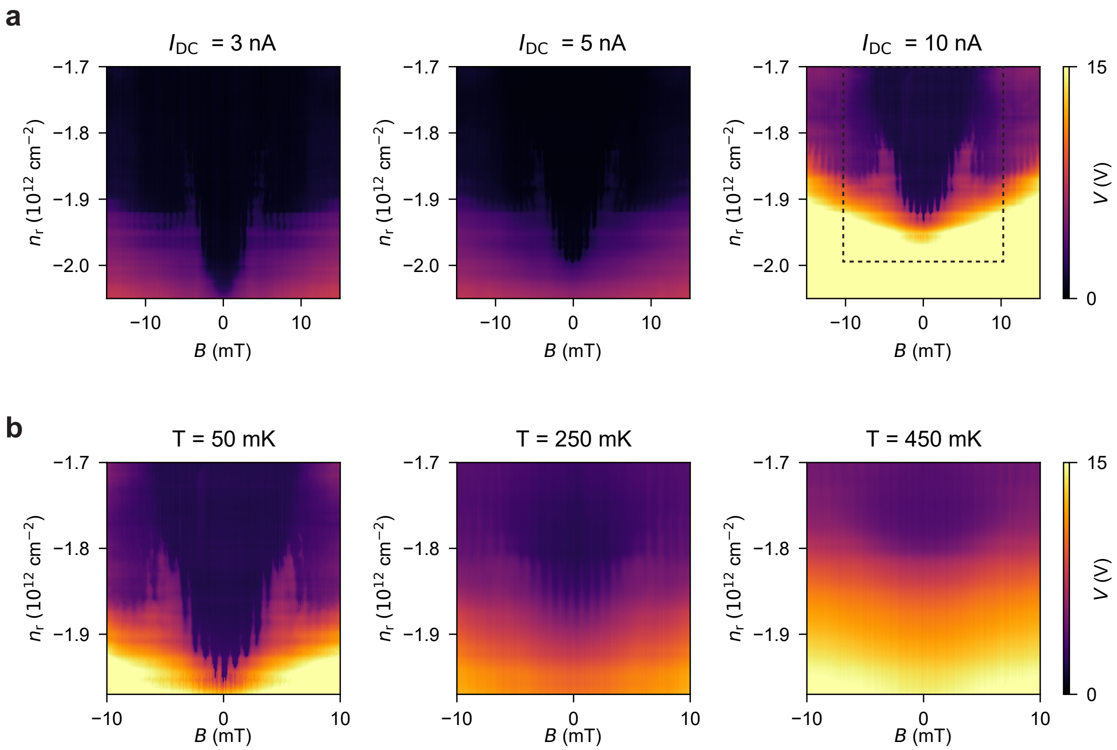

Supplementary Figure 4. **a** Critical density oscillations for $I_{\mathrm{DC}} = 3, 5, 10$ nA at 50 mK. For low I_{DC} (3 nA), the oscillations are not visible at around zero magnetic field. Increasing I_{DC}, oscillations appear starting at zero magnetic field (5 and 10 nA), and the onset density n_{r} of the low resistance region moves towards more negative values. The dashed square in the top right colormap ($I_{\mathrm{DC}} = 10$ nA) indicates the magnetic field and density range of the maps in **b**. **b** Temperature dependence of the critical density oscillations with $I_{\mathrm{DC}} = 10$ nA. Increasing temperature, the extent in n_{r} of the low-resistance region shrinks, and the amplitude of the oscillations increases. The oscillations vanish out at 450 mK.

E. Critical current oscillations in the CI regime

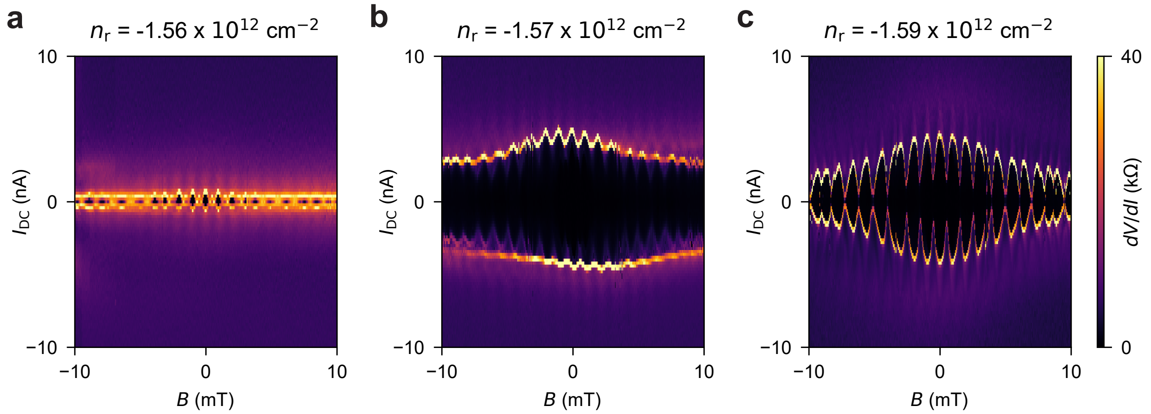

Supplementary Figure 5. **a-c** Critical current oscillations in the (SC, CI) regime, for the ring densities $n_\mathrm{r} = -1.56 \times 10^{12}\mathrm{cm}^{-2}, -1.57 \times 10^{12}\mathrm{cm}^{-2}, -1.59 \times 10^{12}\mathrm{cm}^{-2}$. The same colorbar applies to the three maps. The oscillation period (peak-to-peak distance of critical current) is 1.13 mT and independent of n_r. At a low density ($n_\mathrm{r} = -1.56 \times 10^{12}\mathrm{cm}^{-2}$), a chain of low resistance (superconducting) region appears in between the high resistance (normal conducting) background. The appearance of the oscillations drastically changes as the density is slightly modulated to -1.57 and -1.59 $\times 10^{12}\mathrm{cm}^{-2}$. For those densities, oscillations in resistance are no longer observed for $I_\mathrm{DC} = 0\,\mathrm{nA}$, and only the critical current oscillations appear. We also note that the pattern at $n_\mathrm{r} = -1.57 \times 10^{12}\mathrm{cm}^{-2}$ is largely tilted.

F. Critical current, phase diagram and critical field and temperature of the ring in the CI and BI regimes

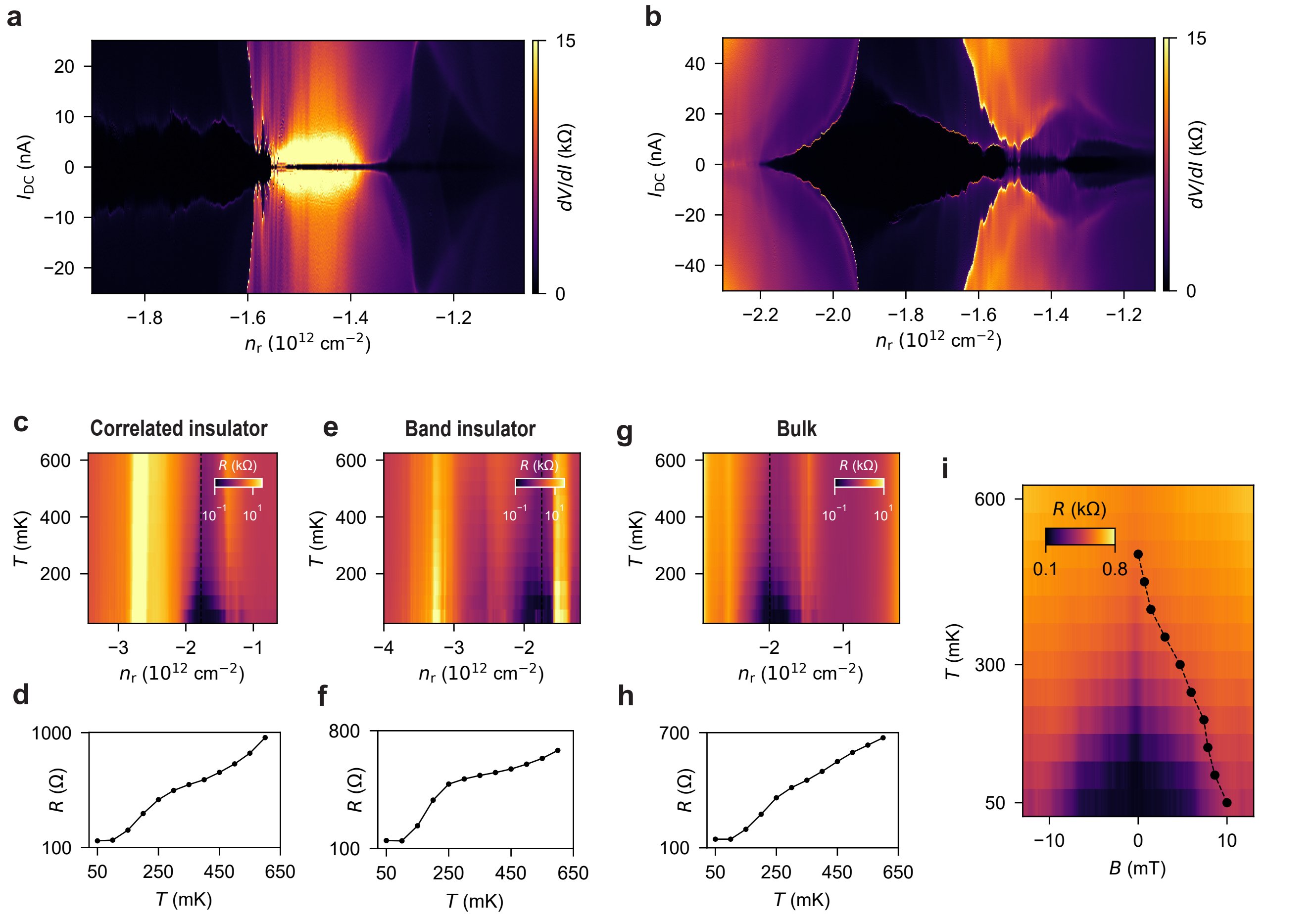

Supplementary Figure 6. **a**, **b**: Differential resistance dV/dI as a function of n_r when $n_g = -1.49 \times 10^{12}$ cm^{-2} (**a**, in the CI state) and $n_g = -3.13 \times 10^{12}$ cm^{-2} (**b**, in the BI state). **c**, **e**, **g**: Phase diagrams of the sample as a function of temperature and carrier density. Three configurations are investigated: **c** the ring surrounded by CI ($n_g = -1.49 \times 10^{12}$ cm^{-2}), **e** the ring surrounded by BI ($n_g = -3.13 \times 10^{12}$ cm^{-2}) and **g** bulk ($V_{rg} = 0$). (**d**, **f**, **h**): Temperature dependence of the resistance at a fixed n_g, in the superconducting dome. The dashed lines in **c**, **e**, **g** show the point at which the data is taken. **i** Temperature and magnetic field dependence of the resistance when $n_g = -2.02 \times 10^{12}$ cm^{-2} ($V_{rg} = 0$ V). The markers show the critical magnetic field B_c at which the resistance increases. By fitting the temperature T dependence of B_c with $B_c(T) = \frac{\phi_0}{\xi_{BCS}^2}(1 - \frac{T}{T_c})$, we estimate the BCS coherence length ξ_{BCS} to be around 160 nm. All the measurements are taken using an AC current of 1 nA.

G. Spectrum of the oscillations in the BI regime

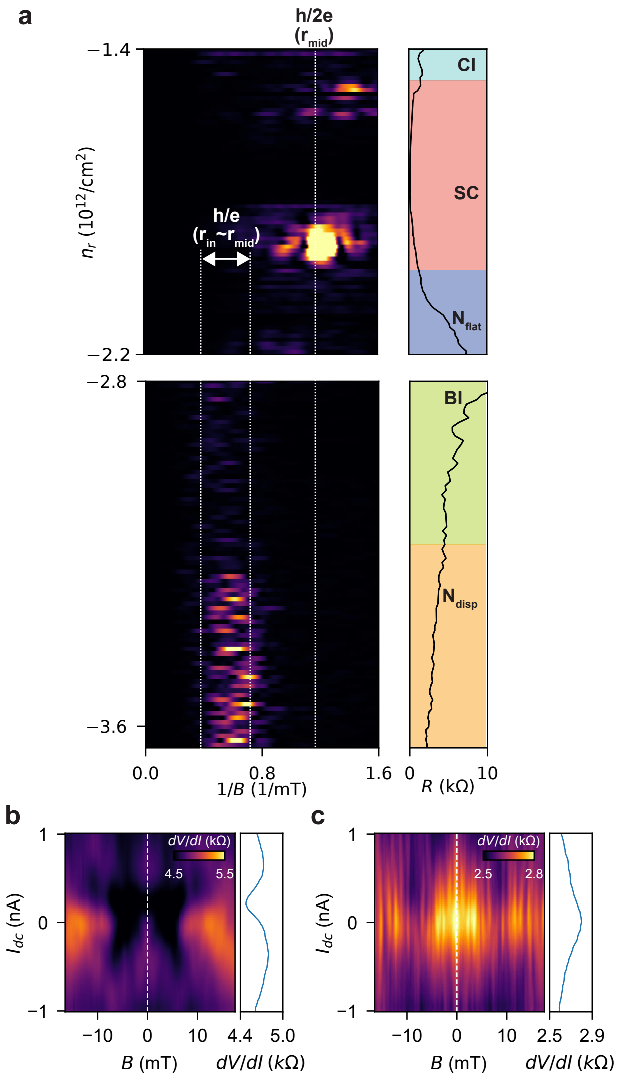

Supplementary Figure 7. **a** Spectrum of the magneto-resistance oscillation with the ring surrounded by BI state. The white-dashed line labelled $h/2e$ (r_{mid}) indicates the frequency corresponding to an $h/2e$-periodicity with $r_{\mathrm{eff}} = r_{\mathrm{mid}}$. The frequency range labelled h/e ($r_{\mathrm{in}} \sim r_{\mathrm{mid}}$), indicates the region with h/e-periodicity with r_{eff} between r_{in} and r_{mid}. **b** Differential resistance as a function of I_{DC} and B for the (N_{flat}, BI) regime (left) and the (N_{disp}, BI) regime (right). The white dashed line indicates $B = 0$. In the side panel of each map, dV/dI is plotted as a function of I_{DC} at $B = 0$.

II. SUPPLEMENTARY DISCUSSION

A. Electrostatic simulation

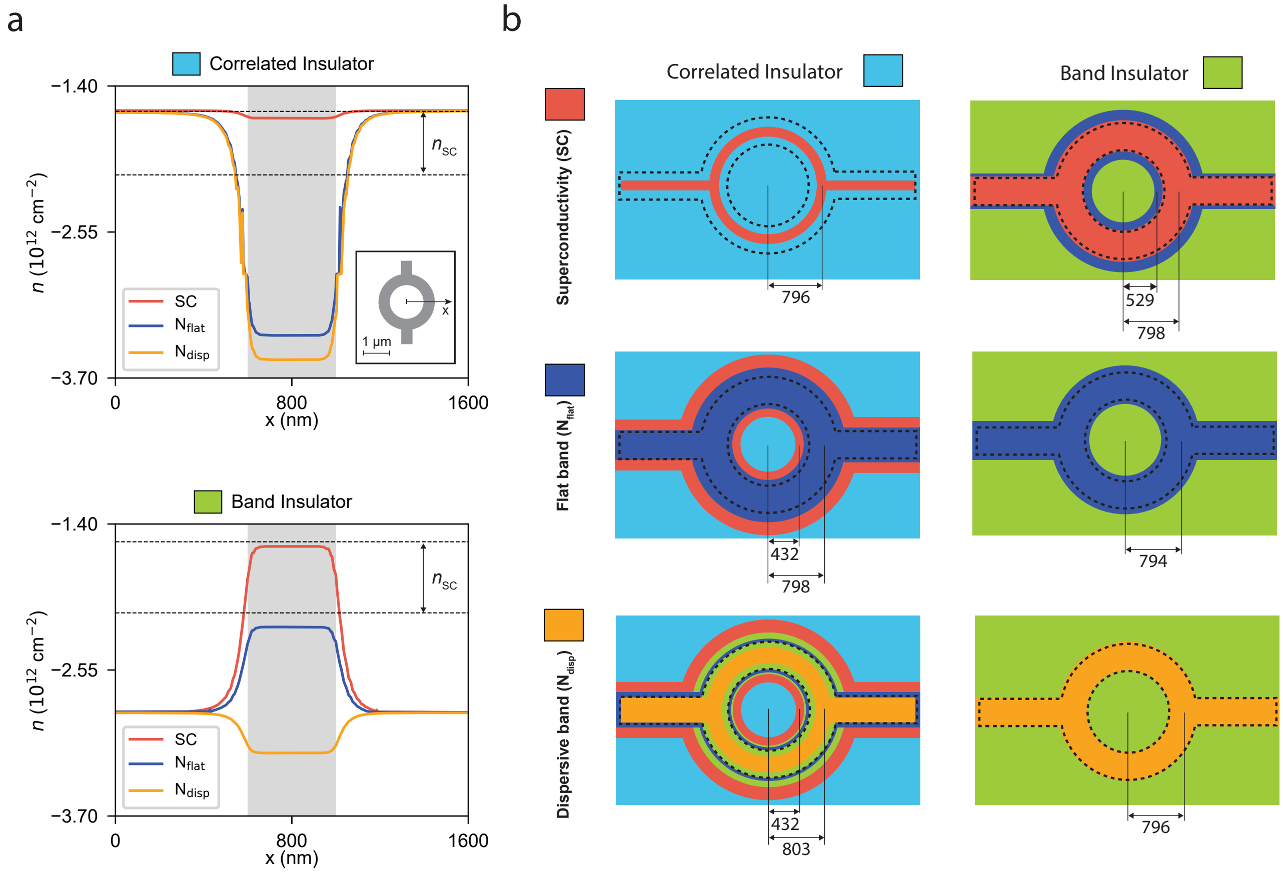

Supplementary Figure 8. Electrostatic simulations of the spatial distribution of the carrier density in the ring. **a** Carrier density distribution along the radial (x) axis. The inset in the top panel shows schematics of the structure and indicates the direction of the x axis. In the top panel, the bulk is set in the correlated insulator (CI) state, and in the bottom panel, in the band insulator (BI) state. For CI and BI cases, we simulate the density distribution with the ring in the superconducting (SC), normal conducting flat band (N_{flat}) or dispersive band (N_{disp}). The gray shaded area indicates the lithographic width of the ring (400 nm), and the horizontal dotted lines are the densities at which the ring is superconducting (n_{sc}). **b** Spatial density distribution and stripe structure of the ring for the six regimes. Numbers with arrows indicate the dimension of each part, in the units of nm.

We perform electrostatic simulations of the carrier density distribution in the MATBG ring presented in this work. The inset in the top panel of Supplementary Figure 8a shows a top view of the geometry of the structure. In the simulations, we discretize the system with a finite element model and self-consistently solve the Poisson equation ($\varepsilon_0 \varepsilon_r(\mathbf{x}) \nabla V(\mathbf{x}) = \rho(\mathbf{x})$) using the software COMSOL Multiphysics. In the equation, ε_0 is vacuum permittivity, ε_{r} is relative permittivity of the dielectric layer ($\varepsilon_{\mathrm{r}}(\mathrm{hBN}) = 3.5$ and $\varepsilon_{\mathrm{r}}(\mathrm{AlOx}) = 9.5$), $V(\mathbf{x})$ is electric potential at position $\mathbf{x}$, and $\rho(\mathbf{x})$ is the charge density at position $\mathbf{x}$. We impose $\rho(\mathbf{x}) = 0$ in the dielectric layers and implement $\rho(\mathbf{x})$ in the MATBG plane using the Thomas-Fermi approximation[1]. We simplify the band structure of MATBG assuming parabolic bands for both the flat and the dispersive bands, with respective effective masses $m_{\mathrm{flat}} = 0.05 m_{\mathrm{e}}$ and $m_{\mathrm{disp}} = 0.4 m_{\mathrm{e}}$[2,3]. We assume a band gap of 30 meV for the BI state, separating the flat and dispersive bands[3]. We impose $V = V_{\mathrm{rg}}$ on the top side of the top hBN layer and $V = V_{\mathrm{bg}}$ at the bottom of the bottom hBN layer. We approximate the CI state with a smaller energy gap of 0.3 meV. After solving Poisson's equation, the carrier density $n(\mathbf{x})$ is directly extracted from the calculated surface charge density $\rho_{\mathrm{S}}(\mathbf{x})$ using $\rho_{\mathrm{S}}(\mathbf{x}) = -en(\mathbf{x})$.

The simulated carrier density distributions along the horizontal cut across the arm of the ring are shown in Supple-

mentary Figure 8a The left and right panels correspond, respectively, to the regimes in which the outside of the ring is in the CI ($V_{\mathrm{bg}} = -4.5$ V) and BI regime ($V_{\mathrm{bg}} = -8.2$ V). In both panels, we show the charge distribution when the ring is set to the superconducting state ($V_{\mathrm{rg}} = -0.065$ V when CI outside and $V_{\mathrm{rg}} = 1.69$ V when BI outside), normal conducting in the flat band ($V_{\mathrm{rg}} = -2.31$ V when CI outside and $V_{\mathrm{rg}} = 0.88$ V when BI outside) and in the dispersive band ($V_{\mathrm{rg}} = -2.55$ V when CI outside and $V_{\mathrm{rg}} = -0.44$ V when BI outside). The vertical dashed lines indicate the lithographic width of the ring (400 nm), while the horizontal lines show the density range of the superconducting state. For all six conditions, the fringing field results in a broadening of the carrier density distribution, making the actual width of the targeted quantum state wider/smaller than the lithographic width of the ring gate. Supplementary Figure 8b shows the carrier density distribution in the MATBG layer for the six configurations. Because the quantum states in MATBG are density-dependent, the broadening of the carrier density distribution leads to a stripe structure of phases across the sample. The insets in Supplementary Figure 8b show the quantum state distribution across the arm of the ring. The charge densities at which each quantum state (CI, SC, Nflat) arises are extracted from the map in Fig. 1.b. Owing to the circular symmetry of the system, multiple quantum state stripes form a ring with different radii and could contribute to oscillations.

Even in ($\mathrm{N_{flat}}$, CI) regime, a superconducting path can be formed. In particular, there is a small superconducting ring with a radius of 432 nm and an open path surrounding the outer edge of the $\mathrm{N_{flat}}$ region. Such a spurious superconducting path could contribute to the non-linear transport characteristics such as the one observed in Fig. 3c. Moreover, the oscillations in resistance and critical current observed in the ($\mathrm{N_{flat}}$, CI) regime have a period of 3.19 mT, corresponding to an effective radius of 446 nm in $h/2e$. This number coincides with the small superconducting ring predicted by the simulation, though more work is necessary to confirm the existence of such a ring by taking the superconducting proximity effect into account.

Such a satellite superconducting path is also predicted in the ($\mathrm{N_{disp}}$, CI) regime, where only the conventional Aharonov–Bohm effect (r_{eff} matches r_{in} and r_{mid}) and no superconducting-like non-linearity are experimentally observed. In this regime, there is a BI state between the $\mathrm{N_{disp}}$ state and the satellite superconducting path, which might make the majority of the current flow away from the superconducting path.

B. Tunable phase shift of the Little–Parks effect

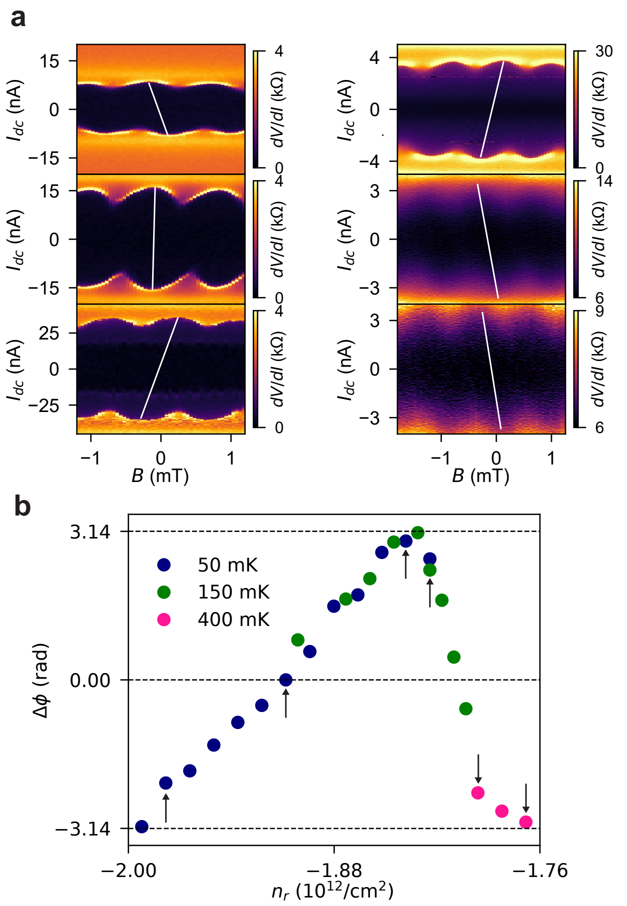

Supplementary Figure 9. **a** Detailed evolution of the asymmetry with n_r. The white lines connect the maximum critical current at positive and negative I_DC. **b** Phase-shift $\Delta\Phi$ as a function of n_r at 50, 150 and 400 mK.

The magnetic field at which the critical current reaches its maximum (B_max) depends on the sign of the current, as can be seen in Supplementary Figure 9a. This means that there is a phase shift in the magneto-oscillation of the critical current depending on the polarity of the bias current. Here, we show that the ring gate tunes this phase shift. To characterize this phase shift, we extract $\Delta B = B_\mathrm{max}(I > 0) - B_\mathrm{max}(I < 0)$ and convert it to a phase shift in flux $\Delta\phi = \frac{\Delta B \times \pi (R_\mathrm{eff})^2}{h/2e}$. As shown in Supplementary Figure 9b, $\Delta\phi$ monotonically decreases from π to $-\pi$ and vanishes when $n_\mathrm{r} = -1.90 \times 10^{12}\mathrm{cm}^{-2}$.

Such an asymmetry can be attributed to the slight shape-asymmetry of the superconducting path inside the ring, which is presumably gate-dependent. It could result in a difference in the inductance of each arm.

Our observations imply that special care has to be taken when analyzing the phase of the Little–Parks oscillations in critical current in thin superconductors, as the large inductance of the material can result in an inevitable phase shift.

C. Interpretation of the temperature dependence of the Little–Parks effect

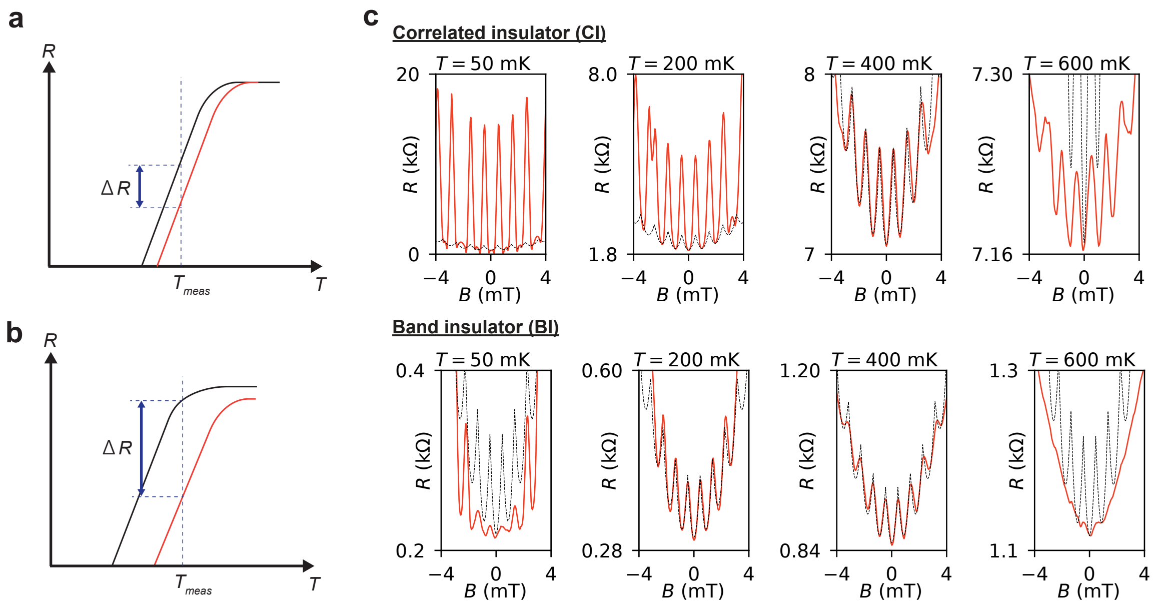

Supplementary Figure 10. Illustration of the temperature dependence of resistance of a ring with (black) and without (orange) magnetic field. **a** The ideal case where the shape of the temperature dependence does not depend on the magnetic field, namely R(T). **b** The case with magnetic field dependence, namely R(T,B). **c**: Results of the fitting of the data to Tinkhams's formula for the (SC, CI) regime (top panel) and (SC, BI) regime (bottom panel).

The Little–Parks effect is usually measured as a magneto-resistance oscillation and then converted to an oscillation of the critical temperature. There, a translation of $\Delta R = \frac{dR}{dT} \times \Delta T_{\rm c}$ is used (see Supplementary Figure 10(a). Here, ΔR and $\Delta T_{\rm c}$ are the amplitude of the magneto oscillation of the resistance and the critical temperature, respectively. To compare the theoretical curve for Little-Parks oscillations and the experimental data, we used the following formula: $\Delta R = \frac{dR}{dT} T_c \left[(\frac{\xi_0}{R})^2 (N - \frac{\phi}{\phi_0})^2 + (\frac{\xi_0 w}{\sqrt{3}\phi_0})^2 B^2 \right]$, where $T_{\rm c}$ is the critical temperature at zero magnetic field, ξ_0 is the the BCS coherence length, N is the integer, and w is the ring's width[4,5]. To plot the theoretical curve, we took the lithographic width of the ring (w = 400 nm). Here, it is assumed that the shape of the temperature dependence of the resistance $R(T)$ does not change with the magnetic field, making ΔR constant in a wide range of temperature as shown in Supplementary Figure 10a. However, as shown in Supplementary Figure 10, the Little–Parks effect observed here strongly depends on the temperature. In addition, as we show here, fitting the magneto-resistance data to Tinkham's formula is valid in a limited range of temperatures even though the observed amplitude of ΔR is larger than the expected value by a factor of ten. Such discrepancies could arise from the breakdown of the assumption that the shape of the temperature dependence of the resistance does not change with the magnetic field. In 2D superconductors such as MATBG, the thickness is always shorter than the penetration length of the magnetic field (Pearl length), letting magnetic flux always penetrate as soon as the magnetic field is applied. Therefore, the critical temperature is modulated not only by the Little–Parks effect but with the magnetic field itself, making Tinkham's assumption break. In addition, the ring's width can deviate from the lithographic one in at least two ways. One is the broadening of the effective width of the ring due to the fringing field effect. The values from the simulation depart from the lithographic one by a factor of < 1.5. However, since the simulation does not take the temperature effect into account, the comparison between the theoretical curve and the experimental data remains qualitatively the same as the one shown in the Figure. The other mechanism that may affect the ring's width is the superconducting proximity effect, which enhances the width of the superconducting region. This mechanism could modulate the effective radius or the width, which depends on temperature. The qualitative estimation of the proximity effect is a challenging problem due to the lack of understanding of the proximity effect between the superconductor and other quantum states in MATBG (e.g., correlated insulator), making this problem open for future efforts.

Below, we discuss that the observation of the Little–Parks effect prefers a temperature that is close enough to the critical temperature. The condensation energy U of a superconductor with a width w, thickness t and length L is

$U = \frac{1}{2\mu_0}(\frac{\phi_0}{2\pi\xi^2})^2 Lwt$. Here, μ_0 is vacuum permeability, and ξ is the Ginzburg-Landau coherence length $\xi = \frac{\xi_0}{(1-\frac{T}{T_c})^{1/2}}$ with ξ_0 being the zero-temperature coherence length. Then, $U < k_B T$ sets an approximate condition that such a piece of superconductor becomes unstable. At a fixed temperature T, the maximum length of such a broken area is

$$L = \frac{2\mu_0 k_B T}{wt}\left(\frac{2\pi\xi^2}{\phi_0}\right)^2. \qquad \text{(Supplementary Equation 1)}$$

Then, if $L < 2\pi R$, the superconductor hosts a piece of normal conducting part, which does not extend across the entire ring. Such a part would make a Josephson junction in the system. Equation Supplementary Equation 1 leads to the condition that

$$k_B T \le \frac{Rwt\phi_0^2}{4\pi\mu_0\xi^4} = \frac{Rwt\phi_0^2}{4\pi\mu_0\xi_0^4}\left(1 - \frac{T}{T_\mathrm{c}}\right)^2. \qquad \text{(Supplementary Equation 2)}$$

Equation Supplementary Equation 2 predicts a characteristic temperature T^* at which the non-superconducting regime extends the entire ring. T^* is analytically obtained

$$T^* = \frac{T_\mathrm{c}(-\sqrt{T_\mathrm{c}(T_\mathrm{c}+4\alpha)} + 2\alpha + T_\mathrm{c})}{2\alpha}. \qquad \text{(Supplementary Equation 3)}$$

Here, $\alpha = \frac{Rwt\phi_0^2}{4\pi\mu_0\xi_0^4}$. It can be seen that T^* is very close to T_c. For example, if $R = 850$ nm, $\xi_0 = 160$ nm, $w = 400$ nm, $t = 0.34$ nm, and $T_\mathrm{c} = 600$ mK, $T^* = 594$ mK. Though the above calculation is a crude estimation, it is suggested that the ring becomes more homogeneous as the temperature increases close to the critical temperature. This supports that the observed asymmetry in the Little–Parks oscillations becomes less prominent at higher temperatures and with a density n_r where the superconducting state has a small critical current.

D. Interpretation of the beating pattern

As shown in Fig. 2e, the critical current reaches zero at $B \sim 5\,\mathrm{mT}$ in the (SC, BI) regime. One explanation of such an effect is the formation of a spurious Josephson junction in the system, that would produce a Fraunhofer-like pattern. However, this hypothesis fails to explain the observed data quantitatively. Namely, for a 2D planar Josephson junction[6,7], the magnetic field at which the critical current vanishes is $\sim 1.8(h/2e)/W^2$, where W is the lateral width of the junction. Using this formula, the magnetic field of $\Delta B \sim 5\,\mathrm{mT}$ is converted to an effective junction width of $W \simeq 860\,\mathrm{nm}$. This dimension significantly exceeds the width of the arm of the ring (400 nm), inferring that the superconductivity inside the ring is homogeneous and does not host any junction.

Another possibility that could explain the vanishing critical current is the existence and interference of multiple superconducting paths inside the ring. In fact, the central frequency of the Little–Parks oscillation ($\sim$1.1/mT) coincides with the center radius of the ring ($\sim$855 nm). If superconducting paths with effective radii of $\sim$87 nm (frequency $\sim$0.8/mT) or $\sim$650 nm (frequency $\sim$1.2/mT) are also present, their interference would produce a beating frequency of 0.2/mT that equals to the beating periodicity of 5 mT.

E. Distinguishing Little–Parks effect from SQUID

Due to the fluxoid quantization in superconductors[8], when a magnetic field is applied perpendicular to the device plane, there will be a circulating current around any hole threaded by the field. If that current flows in a region of the superconductor that has a nonzero resistance this will lead to the so-called Little–Parks effect[9,10]. In the case of 3D superconductors, current flows through the bulk, without encountering any resistance. However, for two-dimensional materials, where the penetration depth of the magnetic field is bigger than the thickness of the material, the whole superconducting surface has nonzero resistance. The Little–Parks effect is thus expected in any two-dimensional superconducting device.

The question that remains in the case of a two-dimensional ring is whether there can be spontaneous formation of Josephson junctions leading to a SQUID-like behaviour. To discern between the two behaviours, we focus on the oscillations in voltage drop across the device when biased *above* the critical current. There, oscillations in the voltage drop are expected both in the Little–Parks and SQUID cases. In the former, they are expected due to the

finite breadth in current of the superconducting transition. In the case of the latter, the oscillations are expected due to the fact that even if the junctions present in the ring are biased above their critical currents, the superconducting condensate in the rest of the ring is still present and thus superconducting coherence and subsequent interference phenomena still take place[11].

The difference between the two types of oscillations is that for the SQUID ones a particular shape is expected while the ones stemming from Little–Parks effect are not expected to take any particular shape. For the ones originating from SQUID behaviour, the following voltage dependence is expected in the case of two overdamped junctions:

$$V = (R/2)\sqrt{I^2 - (2I_\mathrm{c}\cos(\pi\Phi/\Phi_0))^2} \quad \text{(Supplementary Equation 4)}$$

where $R/2$ is the resistance of the two Josephson junctions in parallel, Φ the flux threading the loop, and Φ_0 the superconducting flux quantum. Assuming that the eventual junctions that form in the material are overdamped is reasonable taking into account the low critical currents and quasiparticle resistances expected in junctions in this material, from measurements performed in devices with electrostatically engineered junctions[12]. Supplementary Figure 11 shows the expected voltage drop for such a case. Supplementary Figures 12-14 show the corresponding line plots taken at different current biases when sweeping the magnetic field. We observe no particular resemblance between the data and the expected trend. As a comparison, we performed the same analysis for a SQUID device in this material[12], where the resemblance was clear. Thus, we conclude that it is safe to consider the oscillations as stemming mostly from the Little–Parks effect.

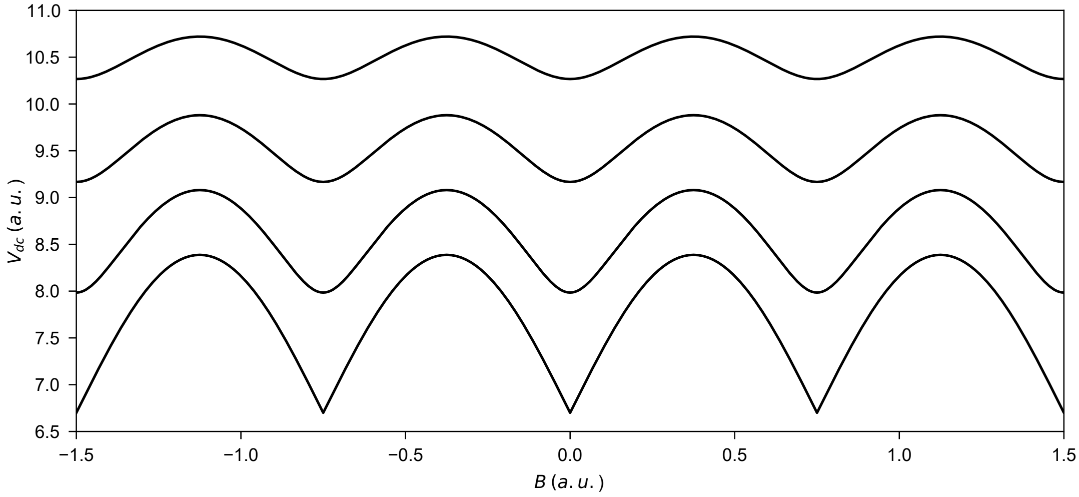

Supplementary Figure 11. Expected voltage drop across a SQUID device with overdamped Josephson junctions as a function of magnetic field. Each solid line corresponds to a different bias current. There is an arbitrary offset for visibility purposes. A higher offset corresponds to a higher current bias.

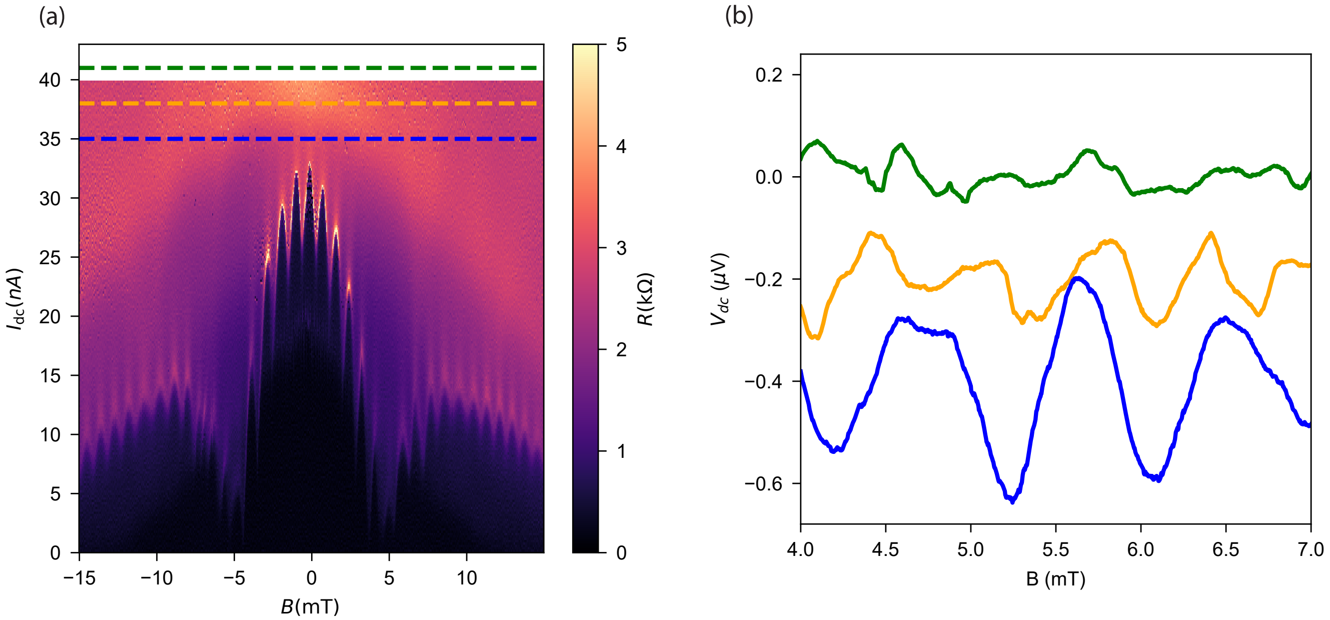

Supplementary Figure 12. **a** Resistance across the device as a function of magnetic field and current bias. The colored dashed lines correspond to the traces shown in Figure (b). **b** Line cuts of Figure a where the voltage drop across the device is shown as a function of magnetic field at different current biases. The value of the top gate voltage at which this data was taken is $V_{\rm rg} = 1.30\,{\rm V}$.

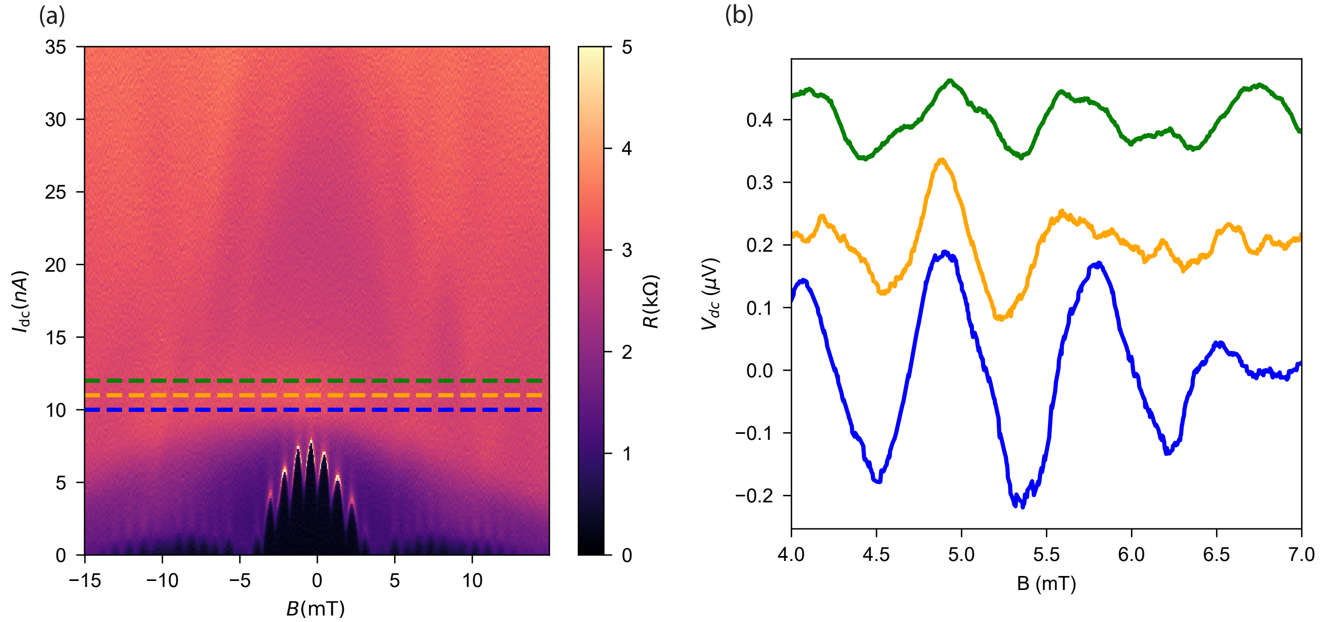

Supplementary Figure 13. **a** Resistance across the device as a function of magnetic field and current bias. The colored dashed lines correspond to the traces shown in Figure (b). **b** Line cuts of Figure (a) where the voltage drop across the device is shown as a function of magnetic field at different current biases. The value of the top gate voltage at which this data was taken is $V_{\rm rg} = 1.14\,{\rm V}$.

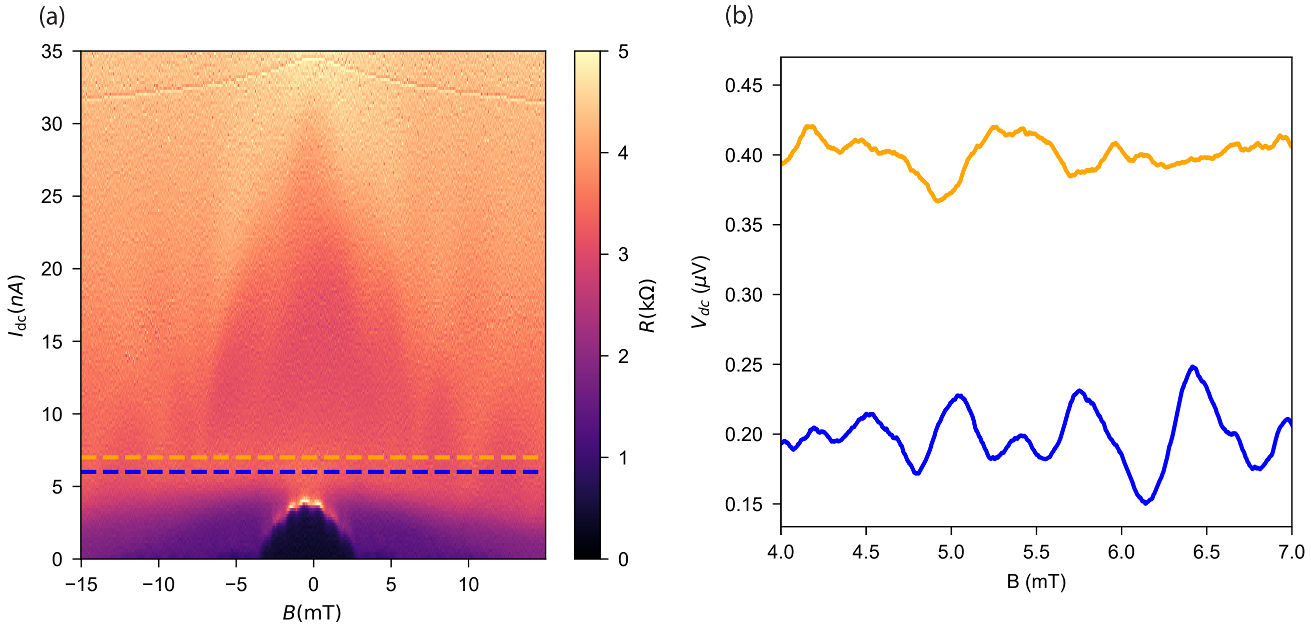

Supplementary Figure 14. **a** Resistance across the device as a function of magnetic field and current bias. The colored dashed lines correspond to the traces shown in Figure (b). **b** Line cuts of Figure (a) where the voltage drop across the device is shown as a function of magnetic field at different current biases. The value of the top gate voltage at which this data was taken is $V_{\mathrm{rg}} = 1.05\,\mathrm{V}$.

SUPPLEMENTARY REFERENCES

[1] Luscombe, J. H., Bouchard, A. M. & Luban, M. Electron confinement in quantum nanostructures: Self-consistent poisson-schrödinger theory. *Phys. Rev. B* **46**, 10262–10268 (1992).

[2] Cao, Y. *et al.* Unconventional superconductivity in magic-angle graphene superlattices. *Nature* **556**, 43–50 (2018).

[3] Rodan-Legrain, D. *et al.* Highly tunable junctions and non-local Josephson effect in magic-angle graphene tunnelling devices. *Nat. Nanotechnol.* **16**, 769–775 (2021).

[4] Moshchalkov, V. V. *et al.* Effect of sample topology on the critical fields of mesoscopic superconductors. *Nature* **373**, 319–322 (1995).

[5] Tinkham, M. Effect of fluxoid quantization on transitions of superconducting films. *Phys. Rev.* **129**, 2413–2422 (1963).

[6] Clem, J. R. Josephson junctions in thin and narrow rectangular superconducting strips. *Phys. Rev. B* **81** (2010).

[7] Fermin, R., De Wit, B. & Aarts, J. Beyond the effective length: How to analyze magnetic interference patterns of thin-film planar Josephson junctions with finite lateral dimensions. *Phys. Rev. B* **107**, 064502 (2023).

[8] Tinkham, M. *Introduction to Superconductivity* (Dover, 2004).

[9] Little, W. A. & Parks, R. D. Observation of Quantum Periodicity in the Transition Temperature of a Superconducting Cylinder. *Phys. Rev. Lett.* **9**, 9–12 (1962).

[10] Groff, R. P. & Parks, R. D. Fluxoid Quantization and Field-Induced Depairing in a Hollow Superconducting Microcylinder. *Phys. Rev.* **176**, 567–580 (1968).

[11] Clarke, J. & Braginski, A. I. *The SQUID Handbook Fundamentals and Technology of SQUIDs and SQUID Systems*, vol. 1 (Wiley-VCH, 2006).

[12] Portolés, E. *et al.* A tunable monolithic SQUID in twisted bilayer graphene. *Nat. Nanotechnol.* **17**, 1159–1164 (2022).
